# Organiational Structure and Created Values. Review of Methods of Studying Collective Intelligence in Policymaking

**DOI:** 10.3390/e23111391

**Published:** 2021-10-24

**Authors:** Rafał Olszowski, Piotr Pięta, Sebastian Baran, Marcin Chmielowski

**Affiliations:** 1 Faculty of Humanities, AGH University of Science and Technology, Gramatyka 8a, 30-071 Kraków, Poland; pipieta@agh.edu.pl; 2 Center for Collective Intelligence, Massachusetts Institute of Technology, 245 First Street, E94, Cambridge, MA 02142, USA; 3 Department of Mathematics, Cracow University of Economics, Rakowicka 27, 31-510 Kraków, Poland; sebastian.baran@uek.krakow.pl; 4 Fundacja Wolności i Przedsiębiorczości, Ul. Asnyka 6, 40-696 Katowice, Poland; chmielowski@fundacjawip.org

**Keywords:** collective intelligence, crowdsourcing, policymaking, public policy, e-participation, literature review

## Abstract

The domain of policymaking, which used to be limited to small groups of specialists, is now increasingly opening up to the participation of wide collectives, which are not only influencing government decisions, but also enhancing citizen engagement and transparency, improving service delivery and gathering the distributed wisdom of diverse participants. Although collective intelligence has become a more common approach to policymaking, the studies on this subject have not been conducted in a systematic way. Nevertheless, we hypothesized that methods and strategies specific to different types of studies in this field could be identified and analyzed. Based on a systematic literature review, as well as qualitative and statistical analyses, we identified 15 methods and revealed the dependencies between them. The review indicated the most popular approaches, and the underrepresented ones that can inspire future research.

## 1. Introduction

The phenomenon of collective intelligence (CI), which is understood as an ability of a particular collective to solve problems, mainly through gathering data, generating ideas and making decisions, has been the subject of interest of many scientific disciplines in recent years. The primary characteristic of a collective showing a high CI level is its capability to solve problems in which the difficulty exceeds the capacity of an individual. CI frequently manifests itself when cooperation, competition or mutual observation gives rise to totally new solutions to the problems or leads to an increase in the ability to solve them. Contemporary studies on CI, although clearly inspired by the development of the Internet in their origins, have so far been carried out in very diverse disciplines, from biology, through social sciences and organization management, to artificial intelligence.

Several empirical studies and theoretical simulations have proven that a collective can, under certain conditions, achieve better results in problem solving than a narrow group of experts [1,2,3,4,5]. To date, this phenomenon has been studied both as a feature of small groups, in which ties and interactions between participants are strong and the deliberation processes lead to informed intellectual outputs [6,7], and as a statistical phenomenon resulting from the aggregation of a vast number of dispersed opinions coming from incoherent crowds [8,9]. The most promising examples of recent projects in which a high level of CI was observed have combined humans and machines, organizations, and ICT networks [3]. The current empirical studies on CI are therefore largely focused on interactions between users in online communities. In parallel, theoretical work has been carried out to simulate collective behavior with the use of computational methods. One of the most interesting is the approach called swarm intelligence (SI), which takes its inspiration from the biological examples provided by social insects such as ants, termites, bees and flocks of birds. In this model, self-organization takes place in decentralized communities in which the logical process is multi-threaded, chaotic and parallel; in which the threads intertwine and interlock; and in which the agents exhibit adaptive behavior, while also maximizing the number of diverse future paths among the possible choices. Simulations show the possible effectiveness of such a decision model, but its application to real social processes is not easy [10,11,12].

The domain of policymaking (i.e., formulating public policies), which used to be strictly limited to small groups of specialists, is now increasingly opening up to the participation of wide collectives, which are not only influencing government decisions, but also enhancing citizen engagement and transparency, improving service delivery and gathering the distributed wisdom of diverse participants [13,14,15,16]. National and local governments use CI methods in the policymaking processes, such as in legislative reforms [17,18], urban strategy planning [16], analyzing large amounts of social data to detect patterns and abnormalities [19,20], using dynamic models for learning, adaptation and forecasting of policy formulation [21,22], real-time continuous policy monitoring [15,23], as well as online public debates and consultations [24,25]. Opening policymaking tasks to public participation, fuelled by the theories of participatory democracy [26,27] and the concept of deliberative democracy [28], has found its practical expression in a paradigm shift towards collaborative governance [29,30], in which policy issues are addressed by networks of governmental and non-governmental actors. However, some models of CI, especially those that are characteristic of swarm intelligence, seem to be very difficult to reconcile with the common understanding of policymaking.

Although collective intelligence has become a more common approach to policymaking, the studies on this subject have not been conducted in a systematic way. The methods of studying the theoretical models, the successful case studies, the public sphere domains in which projects can be implemented, the expected results and the factors influencing CI vary greatly depending on the scientific discipline in which they are conducted. Moreover, different research traditions often use alternative terminologies to describe the same phenomena, an example of which is the competitive use of the labels “crowdsourcing” and “collective intelligence”. Furthermore, there has been no scientific literature review regarding the phenomenon of CI in the field of policymaking. Research methods and strategies used in the studies conducted so far have not been systematized either. Nevertheless, we hypothesized that the methods and strategies specific to different types of CI studies in the field of policymaking can be identified and analyzed.

In order to better understand the present state of knowledge in this field, we raised the main research question (RQ1): what methods and strategies were specific to the studies on collective intelligence in policymaking during the last 10 years? What was the trend in the number of publications by year, and what were the most common concepts that appeared in the studies concerning CI in policymaking?

To supplement the knowledge about the methods and strategies we planned to identify, additional research questions were established:

RQ2: what statistical dependencies occurred between the identified research methods? What dependencies occurred between the research methods and other features of the analyzed studies?

RQ3: in which research areas were the studies conducted? What research methods and strategies were used in the specific research areas?

RQ4: what research methods and strategies were employed in the most influential works and in the topics of special importance for the study of CI in policymaking?

To answer these questions, we conducted a systematic literature review. On this basis, using the grounded theory method, we were able to categorize the identified approaches into a list of 15 methods and strategies and subsequently performed a series of analyses, described later in this article. With the use of statistical analyses, we revealed the dependencies between different study methods, as well as between study methods and other variables. Our cross-sectional analysis has produced interesting results, which may form the foundation for future projects.

## 2. Materials and Methods

To answer the research questions posed, we divided the work into the tasks described below. In order to answer Research Question 1, we adopted the following work plan:

- Task 1.1. Selection of a database of scientific articles to be searched;
- Task 1.2. Search for the studies on collective intelligence in policymaking in the last 10 years, based on selected keywords;
- Task 1.3. Verification of the trend in the number of articles published per year;
- Task 1.4. Search for the most common concepts and terms that appear in the articles;
- Task 1.5. Identification of the methods and strategies of studying CI in policymaking.

**Figure 1:**
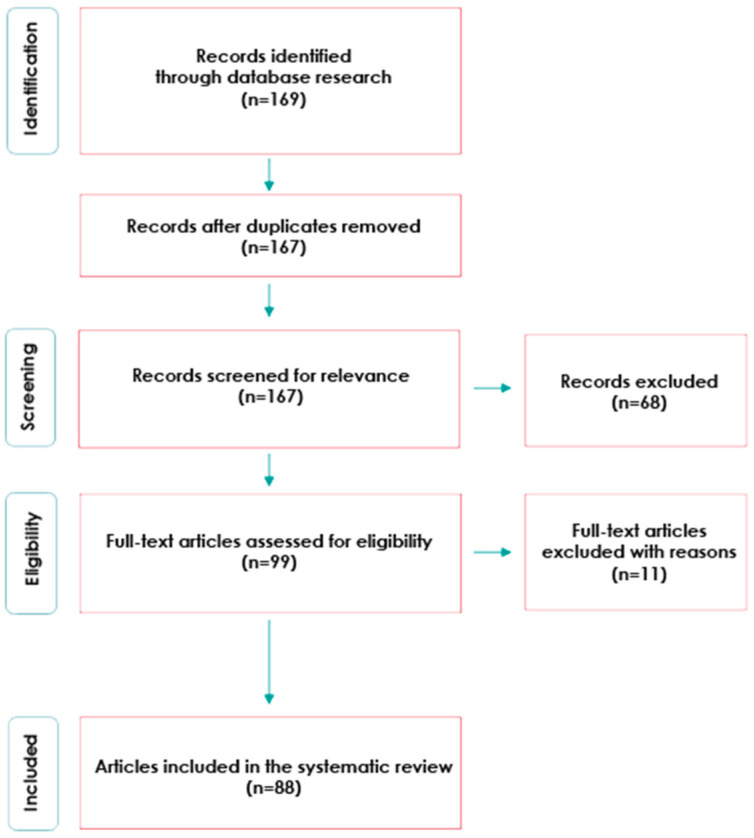
Flow diagram of the article-selection process.

The method used in the first stage of our research was a systematic literature review. This literature review followed the Preferred Reporting Items for Systematic reviews and Meta-analyses (PRISMA) methodology [31]. This section clearly articulates guidelines regarding the inclusion or exclusion criteria of research papers to find relevant papers in our research area. We have also clearly mentioned how and to what extent the review was performed. The PRISMA flowchart for the research process is shown in Figure 1.

When selecting keywords, alternative terms of CI used in the literature were taken into account, including “collective intelligence”, “crowdsourcing”, “swarm intelligence”, “wisdom of crowds” and “crowdlaw”. These concepts, although not fully identical, have an established position, and are used by researchers to describe similar phenomena, depending on the background of individual authors (the relationships and differences between these concepts were described by Buecheler [32]). The second set of keywords included concepts related to political science, administration and governance: “policymaking” (variants: “policy-making” and “policy making”), “public policy”, “political science”, “public administration”, “public sector” and “public governance”. The Web of Science was chosen from a number of pre-selected databases (other databases considered were Scopus, Sciencedirect and EBSCO) because of its reputation for the greatest coverage and the greatest impact in terms of most cited authors and articles, as well as for the most accurate subject classification. Search engines, such as Google Scholar, were excluded, as our priority was to select peer-reviewed publications. The timeframe for the search was set for the period from 2011 to 2020. The data search was conducted on March 8, 2020. We applied the logical search to the topic (including the abstract, keywords and indexed fields), as well as the titles of the scientific articles. The inclusion criteria were focused on peer-reviewed scientific articles dealing with issues in the field of public policymaking and combining them with methods, models and concepts derived from the CI research domain. In addition, we used the language filter to focus on the publications in English.

The logical search used the following syntax: TS = ((“Collective Intelligence” OR “Crowdsourcing” OR “Swarm Intelligence” OR “Wisdom of crowds” OR “Crowdlaw”) AND (“Policy Making” OR “Policy-making” OR “policymaking” OR “Public Policy” OR “Public Administration” OR “Political Science” OR “Public Sector” OR “Public Governance” OR “e-participation”)) OR TI = ((“Collective Intelligence” OR “Crowdsourcing” OR “Swarm Intelligence” OR “Wisdom of crowds” OR “Crowdlaw”) AND (“Policy Making” OR “Policy-making” OR “policymaking” OR “Public Policy” OR “Public Administration” OR “Political Science” OR “Public Sector” OR “Public Governance” OR “e-participation”)).

This search led to an initial total of 169 references, and after removing the duplicates, that number reached 167. Then, in accordance with the guidelines of H. Snyder [33], the content of all articles was screened in terms of checking the inclusion criteria, according to the title-abstract-references scheme, which allowed us to identify the content that did not meet the criteria described above and remove it from the database. To focus on high-quality literature, we excluded the conference proceedings, editorial materials and reviews, and excluded articles written in a language other than English. Another 10 articles were excluded during the eligibility assessment due to the fact that they obviously did not concern the topic of review (e.g., their topic was tourism, citizen science initiatives, the student learning environment, etc.). This led to the refined list of 88 results. By creating the list as described above, it was possible to check how many articles were published annually and what the trends were in the number of publications per year.

The content of the articles was evaluated by our team of 3 experts, with experience and academic backgrounds in both policymaking and information technologies (2 experts with a PhD in political science and experience working on ICT projects, and 1 expert with an MA in IT and experience in working in social projects). The preliminary analysis was made by creating lists of the most common concepts that appeared in article titles, article abstracts, original keywords, as well as KeyWords Plus. The next stage, a qualitative research step, the purpose of which was to extract the methods and strategies of studying CI in policymaking from the analyzed texts, was based on the grounded theory approach. We applied this approach for extracting the theoretical value from the selected studies, grouping and presenting the key concepts, conceptualizing and articulating the concepts and distilling the categories from them. The analysis included stages that were specific to the grounded theory method: open coding, axial coding and selective coding. The open coding stage involved an analytical process of generating high-abstraction level type categories from sets of concepts. In this stage we focused on extracting keywords specific to the analyzed texts that appeared in titles and abstracts. The analysis of keywords allowed for a preliminary division of the texts into 11 subgroups, which became the initial categories. The next stage, i.e., axial coding, aimed to identify the key processes and the main research results described in the examined articles. We adopted an iterative method of working: texts were analyzed in groups of 10, using the existing categories, and then categories were redefined, combined or divided, and their definitions were developed. The emerging categories were grounded during the progressive analysis of subsequent texts from our sample. Then, at the stage of selective coding, the categories were finally integrated and refined [34]. Theoretical saturation was achieved when, during the analysis of the following texts, no new concepts, properties or interesting links arose [35]. Based on the review of the references included in the analyzed texts and the relevant theoretical literature, we adopted the final definitions to describe the identified methods. As a result of the analysis described above, 1 to 5 methods or strategies were identified in each reviewed text, and the general list of 15 methods of studies on CI in the field of policymaking was proposed.

After completing the work described above, we attempted to answer the additional research questions. To answer RQ 2, the following tasks were planned:

- Task 2.1. Checking what number of research methods were used on average per article;
- Task 2.2. Analyzing the changes in the popularity of the use of particular methods in the analyzed period;
- Task 2.3. Finding statistical dependencies between research methods;
- Task 2.4. Finding dependencies between research methods and other features of the analyzed studies (number of citations, usage, number of pages, publication year).

This stage of our research was a series of statistical analyses. The first two tasks were based on the simple counting of averages and the visualization of trends. Then, to analyze the dependencies between research methods, we used Pearson’s Chi-squared test of independence, and Yates’s correction for continuity (Yates’s Chi-squared test). Next, analyzing the dependencies between research methods and other features of the analyzed studies, we had to perform a Shapiro–Wilk test of normality for all continuous variables, the Chi squared of independence test, and statistical analysis based on Pearson’s Chi-squared test of independence. Finally, we used the Fisher exact test of independence.

In order to answer Research Question 3, we planned the following tasks:

- Task 3.1. Identification of the research areas of the studies;
- Task 3.2. Grouping the related research areas, taking into account the specificity of the researched issue;
- Task 3.3. Analysis of the number of studies published yearly within the research area groups;
- Task 3.4. Identification of which methods and strategies of studying CI in policymaking were used more frequently and which were used less frequently within the research area groups.

Based on the WoS Research Areas, we verified in which scientific disciplines the studies were conducted, and what was their number. For the further analytical purposes, we grouped the related scientific disciplines into collections, taking into account the special position of the computer sciences and political sciences. On this basis we tracked the yearly number of studies in each research area group and the most common methods and strategies in each research area.

Finally, to answer Research Question 4, the following tasks were planned:

- Task 4.1 Ranking of the top 10 articles based on usage and citation criteria to identify the most influential works;
- Task 4.2. Identification of which methods and strategies were used more frequently and which were used less frequently in the “top 10” groups;
- Task 4.3. Ranking the topics of special importance for the study of CI in policymaking;
- Task 4.4. Identification of which methods and strategies were used more frequently and which were used less frequently in the “topics of special importance” groups;

To analyze the most influential studies, we ranked the top 10 articles based on usage and citation criteria, obtained from the Web of Science statistics. On this basis we tracked the most common methods and strategies in each research area. Then, building the ranking of topics of special importance, to ensure data triangulation and to avoid duplicating regularities already detected, in the selection of topics we relied on a different method than the one used in the earlier stages of this work. The monographic publications concerning the issues of collective intelligence and policymaking were shortlisted. Due to the scarcity of monographic literature, only 8 publications were included in this list after the review. On this basis, an initial list of 20 concepts was compiled. Subsequently, a survey was conducted in which a group of 6 social science researchers were invited to assess the significance of the proposed issues. Thus, the final list of 7 concepts that were subject to analysis was selected, and we searched our literature database for keywords specific to each of these concepts. The identified sub-groups of studies were analyzed in terms of the research methods and strategies that were adopted.

## 3. Results

### 3.1. Methods and Strategies of Studying CI in Policymaking

#### 3.1.1. Number of Articles in the Selected Database and the Growth Trend

**Figure 2:**
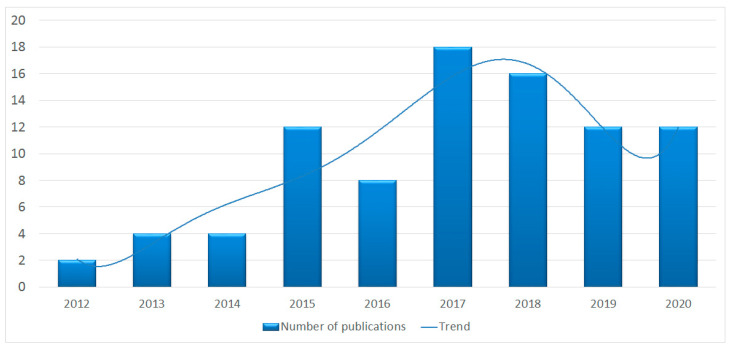
Number of articles concerning the issues of collective intelligence and policymaking published annually, and the growth trend for the period 2012–2020.

As described above, the Web of Science database was selected for our review, and studies were searched within it according to the adopted criteria. After the initial analysis, it was discovered that none of the reviewed articles were published in 2011. The first article that met the inclusion criteria appeared in 2012. In the years 2012–2017, we observed a clear increase in interest in the issue under study. The peak period of interest was 2017, when 18 articles were published. Despite the decrease observed later, 2020 was again characterized by an increase in the number of publications compared to the previous year (see Figure 2 below). 

#### 3.1.2. Concepts and Terms That Appeared in the Articles

We analyzed the content of the research articles included in the review, and created lists of the most common concepts that appeared in article titles, article abstracts, original keywords, as well as KeyWords Plus generated by the Web of Science algorithm [36]. The results are presented below in Table 1.

#### 3.1.3. Identifying Methods and Strategies of Studying CI in Policymaking

In this section, the methods and strategies of studying CI in policymaking, which were identified in the analyzed texts, are presented. As described in Section 2, 15 methods and strategies were identified in the reviewed sample, and each text was associated with a minimum of one and a maximum of five methods. In Table 2 we present a list of identified methods and strategies, ranked from the most to the least popular, and the adopted definitions, supplemented with references to theoretical literature.

As we can see, the analysis of organizational structure/design was the most popular method. Fewer studies used the analysis of created values approach. Subsequent identified methods, such as the analysis of the e-participation process, the analysis of participants’ behavior or collaboration models enjoyed moderate popularity. On the other hand, the least frequently used methods included the analysis of platform usability, analysis of the impact of AI algorithms and analysis of organizational learning. The relatively rare occurrence of the analysis of impact on policymaking approach is also worth noting.

### 3.2. Statistical Analysis

#### 3.2.1. Number of Methods per Article

**Figure 3:**
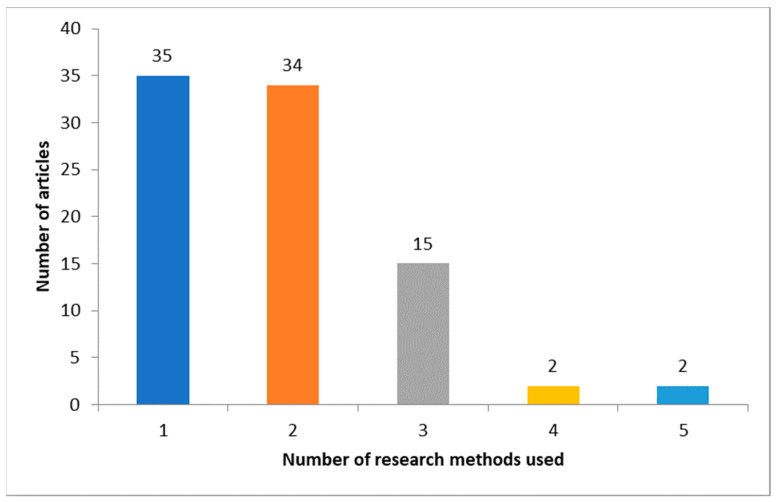
The number of research articles using specified numbers of methods.

On average, 1.89 methods were used per article. Figure 3 visualizes the number of research articles using a specified number of methods. It can be noted that a majority of the analyzed articles used at most two methods.

#### 3.2.2. Changes in the Popularity of Using Particular Methods

**Figure 4:**
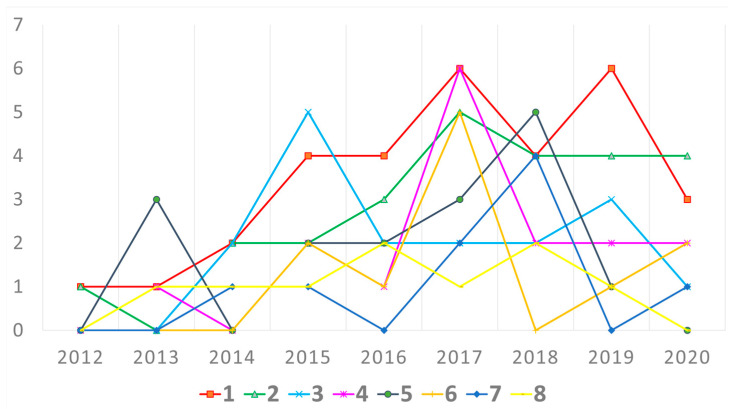
The number of research studies using the following methods: (1) analysis of organizational structure/design, (2) analysis of created values, (3) analysis of e-participation process, (4) analysis of participants’ behavior, (5) analysis of collaboration model, (6) analysis of participants’ motivations, (7) analysis of communication model, (8) analysis of innovation process.

Changes in the number of articles using the identified methods appearing in subsequent years were also analyzed. The chart below shows the yearly numbers of articles using the seven most common methods and strategies, in the period 2012–2020. We can observe that although the analysis of organizational structure has been the most widely used method since 2016, it has recently lost its popularity, falling behind the analysis of created values. In turn, the analysis of the e-participation process, which enjoyed a peak in interest in 2015, has now largely lost its relevance. A similar decline in interest can be observed in relation to the analysis of collaboration model, which peaked in 2018. (as can be seen below in Figure 4).

#### 3.2.3. Dependencies between Research Methods

In this section we answer the question of whether there are any dependencies between the various research methods. It is common that when we want to investigate the relationship between variables, we calculate the classical Pearson’s correlation coefficient. However, Pearson’s correlation coefficient should only be applied to check the dependency between two continuous variables. In our situation this is not the case because the variables describing the usage of research methods are binary variables, answering the question of whether a particular method was used or not. When we are looking for relationships between binary or categorical variables, the commonly used statistical test is Pearson’s Chi-squared test of independence. We performed Pearson’s Chi-squared test between each pair of variables out of all 15 variables, describing the research methods in Table 2. The results can be seen in Table 3.

**Figure A1:**
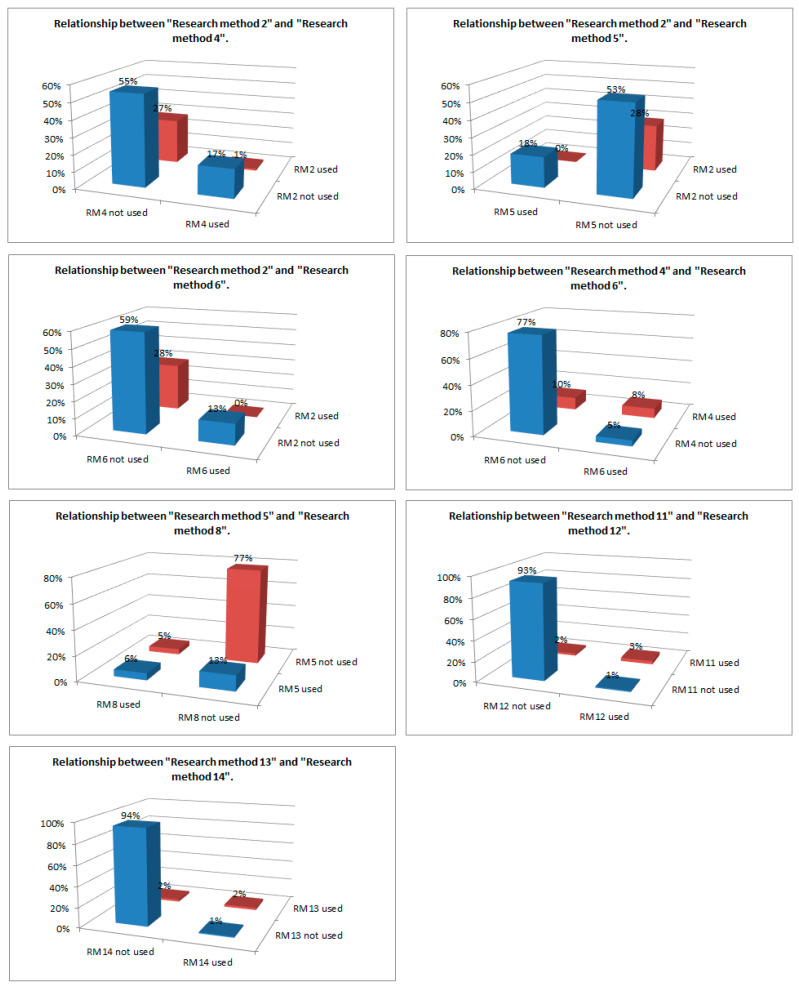
3D barplot of the contingency tables from Table 4 of the Pearson Chi-squared test of independence for the variables with statistically significant dependency.

The statistical analysis based on Pearson’s Chi-squared test of independence showed that in most cases there was no statistically significant evidence of a statistical relationship between research methods (*p*-value > 0.05). The analysis showed that only in seven cases (highlighted in bold in Table 3) was there a significant statistical dependency between certain specific research methods (*p*-value < 0.05). We discuss these dependencies based on the results from Table 4 below and in Figure A1 in the Appendix A.

It must be noted that one of the assumptions of Pearson’s Chi-squared test of independence is the fact that the value of the contingency table cell should be five or more in at least 80% of the cells, and no cell should have a value less than one. Unfortunately, all the contingency tables from Table 4 have at least one cell with a value smaller than five; therefore, the assumption above was not met. Since this was the case, we applied Yates’s correction for continuity (Yates’s Chi-squared test) [59]. The results can be seen in Table 5.

After Yates’s correction there were only five cases with significant statistical dependency between certain specific research methods (*p*-value < 0.05). However, three of them were statistically highly significant (*p*-value < 0.001).

Finally, we can conclude that there are five statistically significant relationships between research method variables: A relationship between analysis of created values and analysis of collaboration model, between analysis of participants’ behavior and analysis of participants’ motivations, between analysis of collaboration model and analysis of innovation process, between categorization of the implemented projects and state-of-the-art review, and finally between analysis of platform usability and analysis of the impact of AI algorithms. Note that the Chi-squared test of independence does not not give an answer as to what kind of dependency exists between variables. It only answers the question of whether there is dependency between variables. To find the limits on what can be shown from the analysis we looked at the contingency tables and corresponding figures and checked if we were able to draw any conclusions from them. From Table 5 and Figure A1 we can suppose that the latter four relationships rely on the fact that in the vast majority of cases, both of these methods were not used simultaneously. In the case of the relationship between analysis of created values and analysis of collaboration model, we can hypothesize that the discontinuation of the analysis of created values method was associated with an increase in the applicability of the analysis of collaboration model method. However, in this case the relationship between variables was not obvious.

#### 3.2.4. Dependencies between Research Methods and Other Features of the Analyzed Studies

**Figure 5:**
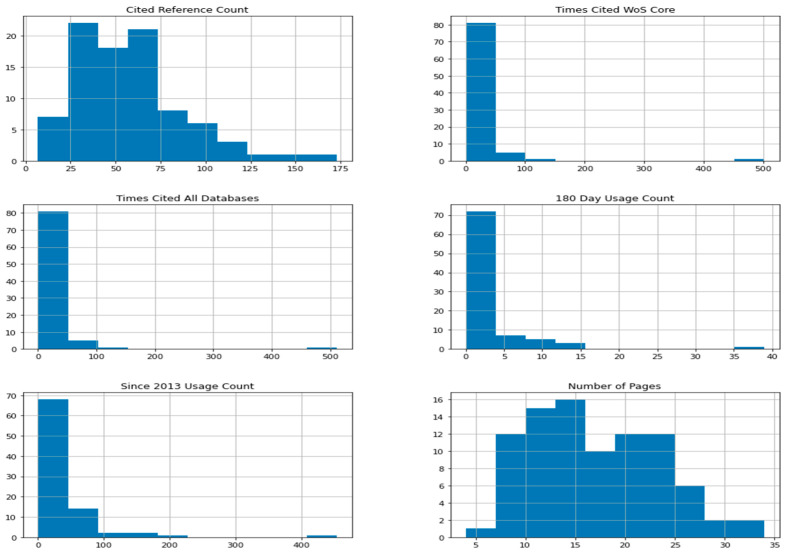
Histograms of the variables: Cited Reference Count, Times Cited WoS Core, Times Cited All Databases, 180 Day Usage Count, Since 2013 Usage Count, Number of Pages.

**Figure 6:**
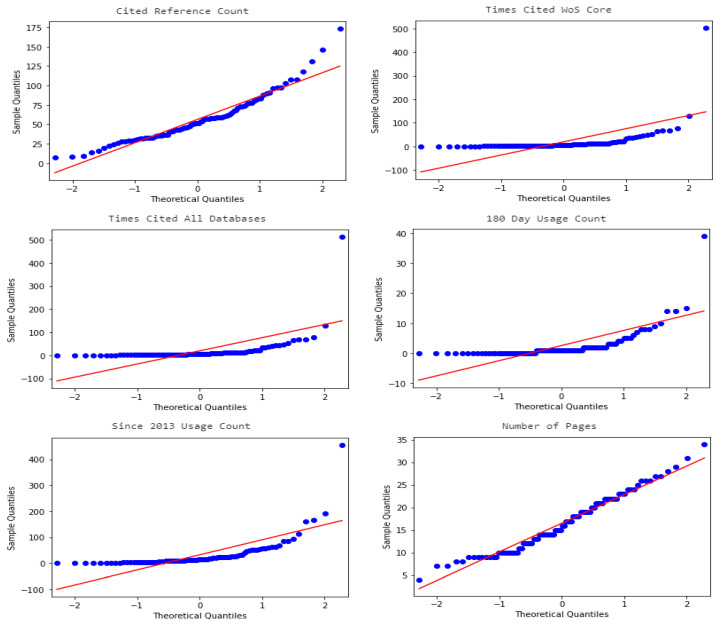
Q–Q plots of the variables: Cited Reference Count, Times Cited WoS Core, Times Cited All Databases, 180 Day Usage Count, Since 2013 Usage Count, Number of Pages.

In this section, we investigate whether there are relationships between the research method used and other article features such as citations, popularity, number of pages and year of publication. As before, in order to perform statistical analysis, we used binary variables describing the use of the peculiar research method in the articles. The variables describing article features are the following: Cited Reference Count, Times Cited WoS Core, Times Cited All Databases, 180 Day Usage Count, Since 2013 Usage Count, Number of Pages and Publication Year (all variables defined in the Web of Science specification [60]). All the above variables except the last one are continuous-type variables and the last one is categorical. In the case of the last variable, the matter is simple. In order to check its relationship with binary variables describing the research methods used, we used the Chi-squared test of independence as before. To check the relationship between binary variables and the other six continuous variables, we calculated the point biserial correlation coefficient. Note that one of the assumptions of the point biserial correlation is the fact that the continuous variable is normally distributed. To check this assumption we plotted histograms, quantile-to-quantile plots and performed the Shapiro–Wilk test of normality for all six continuous variables. The results are shown in Table 6, Figure 5 and Figure 6.

From the histogram plots in Figure 5 we can see that only the distribution of the Number of Pages variable is approximately bell-shaped and therefore looks like a normal distribution. The quantile-to-quantile plots from Figure 6 confirm that only the Number of Pages variable may be normally distributed (because the values are arranged along a straight line). However, if we look at Table 6, we see that the *p*-values of the Shapiro–Wilk test of normality of all the considered variables are small (*p*-value < 0.05) and therefore we must reject the null hypothesis that a sample came from a normally distributed population. Since one of the assumptions of point biserial correlation was not met, we could not use this method to investigate the relationship between binary research method variables and the variables describing article features. However, we used a different solution. We grouped the values of continuous variables into one of three categories: low, medium and high, according to the scheme described in Table 7, and then we used the Chi-squared test of independence as before. The Publication year variable is already a categorical variable. However, due to the fact that it has nine values and the sample size is small, we also grouped its values into three categories. The remaining variables were grouped so that the size of each class was at least 10 and that all classes were more or less equal.

We performed the Chi-squared test of independence for all pairs such that the first variable in the pair was a binary variable describing the research method used and the second variable in the pair was a continuous variable describing the features of the article. The results of the analysis are shown in Table 8.

**Figure A2:**
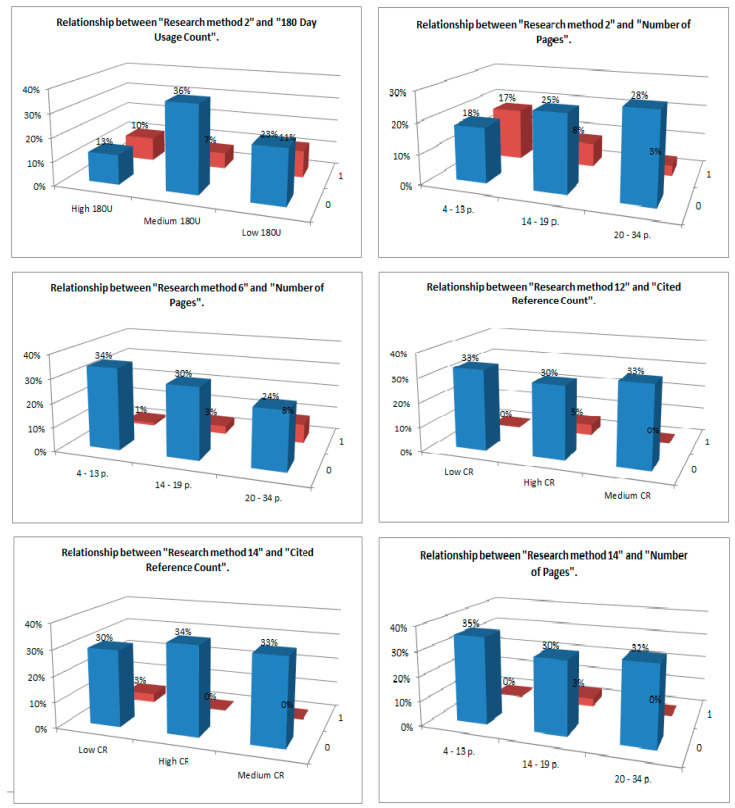
3D barplot of the contingency tables from Table 9 of the Pearson Chi-squared test of independence for the variables with statistically significant dependency.

The statistical analysis based on the Pearson’s Chi-squared test of independence showed that in most cases there is no statistically significant evidence that there is a statistical relationship between research methods and article features (*p*-value > 0.05). The analysis showed that only in six cases (highlighted in bold in Table 8) there is a significant statistical dependency between certain specific research methods and article features (*p*-value < 0.05). We discuss these dependencies based on the results from Table 9 below and in Figure A2 in Appendix A.

For the two first relationships from Table 9, we have enough value in each cell of the contingency table so we can conclude that there is a statistical relationship between the Analysis of created values method and 180 Day Usage Count variable—the use of this method translates into popularity among readers. There is also a statistical relationship between the Analysis of created values method and the number of pages of the article. In this case, it is easy to see from the chart that the use of this research method is related to the reduction of the number of pages of the article in which this method is used.

Unfortunately, the other four contingency tables from Table 9 have at least one cell with value smaller than five; therefore, we should apply Yates’s correction for continuity. However Yates’s correction for continuity is mainly applied for 2 × 2 contingency tables. This is not our case, so we have to use a different statistical test to resolve the remaining four cases. We performed Fisher’s exact test, which is also commonly employed when sample sizes are small or the data are very unequally distributed among the cells of the contingency table. The results of the Fisher exact test can be seen in Table 10.

From the Fisher exact test, it follows that there are two more statistical relationships (*p*-value < 0.05) between the analysis of participants’ motivations method and the number of pages of the article, the state-of-the-art review method and the cited reference count. Again from Table 9 and Figure A2, we can draw some conclusions. It appears that use of the analysis of participants’ motivations method is related to the increase of the number of pages of the article. Moreover, it seems that use of state-of-the-art review method has a positive impact on Cited Reference Count.

The last relationship we looked for was the relationship between the number of methods used in the articles and the features of the article. As before, we conducted the Chi-squared test of independence. For the purposes of the analysis, the variable describing the number of methods used was divided into four categories: one method, two methods, three methods, and 4–5 methods. Due to the small number of the articles with four or five methods used, these articles were grouped into one category. The obtained results (compare Table 11) showed that there is no statistically significant evidence that there was a statistical relationship between the number of methods used in the articles and the features of the article (*p*-value > 0.05).

### 3.3. Research Areas

#### 3.3.1. Identification of the Research Areas of the Studies

**Figure 7:**
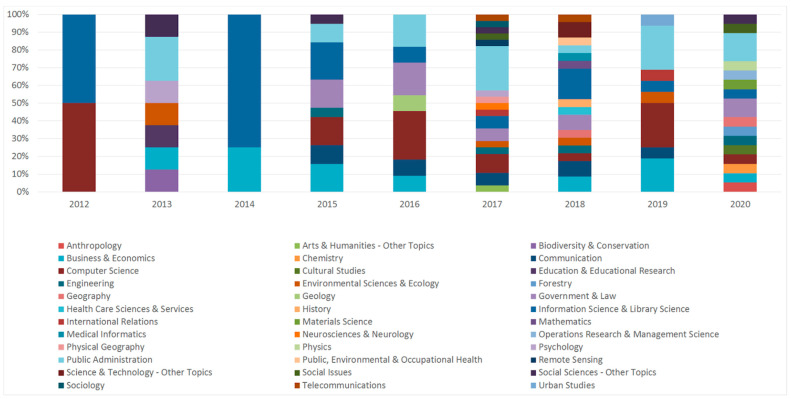
The WoS Research Areas assigned to the journals publishing the analyzed texts (percentage per year).

When classifying the research areas to which the analyzed texts belonged, we used the WoS Research Areas label, which was assigned to each journal publishing the analyzed texts (every record in the Web of Science core collection contains the subject category of its source publication, assigned to at least one of the subject categories). Figure 7 shows in which WoS Research Areas the texts were published in the analyzed period.

We observe that in the first year covered by the analysis (2012), the studied texts belonged to only two research areas, which also happened to be closely related (i.e., information science and computer science), whereas in the subsequent years (with the exception of year 3) the number of research areas systematically grew, reaching its peak in 2017 (17 research areas), and almost maintaining this high level in 2018 and 2020 (16 research areas).

#### 3.3.2. Grouping the Identified Research Areas

For the sake of the clarity of the analysis, we have grouped the emerging research areas into five research area groups (RAGs) as shown in Table 12. We have paid special attention to two general areas that we found of particular importance to the studied topic, here separated into broad categories: (1) computer science, information science and related and (2) political sciences and related. Other research areas in which the references to CI in policymaking appeared were gathered into three groups: (3) humanities and social sciences other than political sciences, (4) natural sciences and mathematics, and (5) applied sciences. In some cases, one article was assigned to more than one research area because it belonged to multiple disciplines according to the Web of Science classification. This was the case for 32 articles of 88 analyzed.

#### 3.3.3. Studies Published Yearly within the Research Area Groups

**Figure 8:**
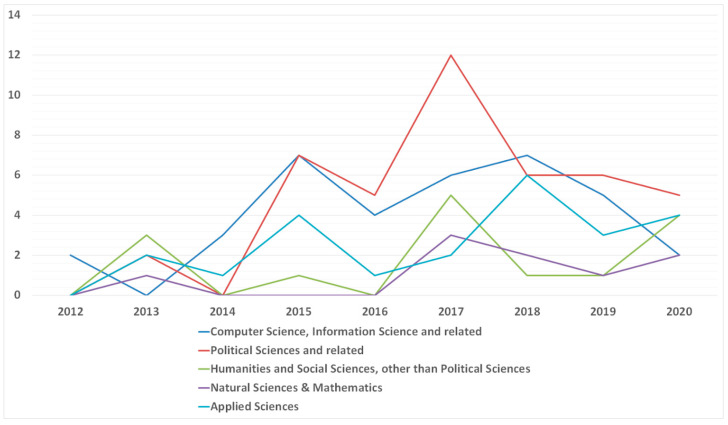
The number of studies on collective intelligence in policymaking published yearly within the RAGs.

The next stage of the work was an analysis of the number of studies published yearly, within the research area groups. This revealed that until 2017 computer science and related was the leading approach. However, since 2017, political sciences have become the main field of research in which studies on collective intelligence in policymaking are conducted. In recent years, the amount of research conducted in the field of computer science has clearly decreased, giving way to various types of social research. Changes in the amount of work published annually within the grouped research areas are shown in Figure 8.

#### 3.3.4. Study Methods Used within the Research Area Groups

**Figure 9:**
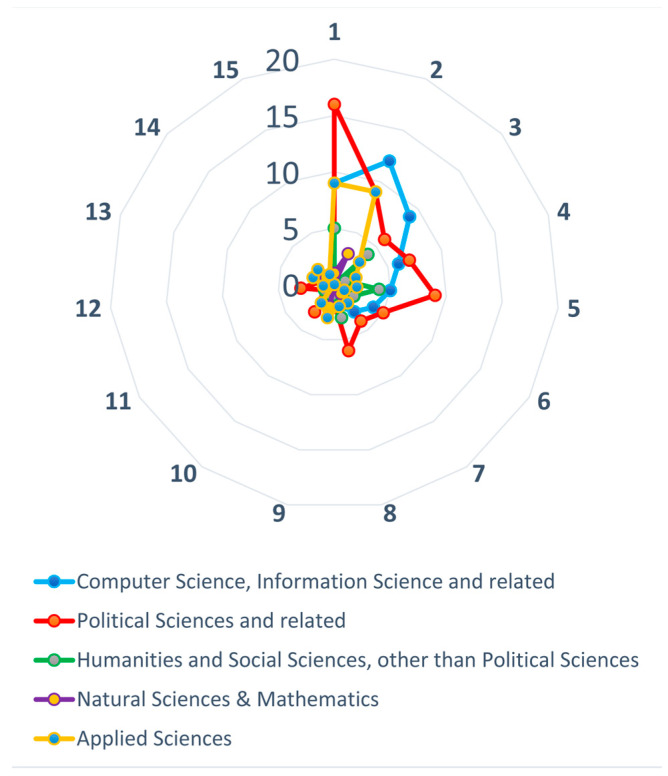
Number of research articles in which the methods and strategies used for studying CI in policymaking were used, broken down by research area groups, in total for the period 2012–2020. The assignment of particular methods and strategies to the labels numbered from 1 to 15, as described in Table 2.

The next stage of our work was to verify, based on the texts that were analyzed, which methods and strategies of studying CI in policymaking were used in the research areas. Figure 9 visualizes the number of research articles, in which the specific methods and strategies used for studying CI in policymaking were used, broken down by research areas, in total for the period 2012–2020.

**Figure 10:**
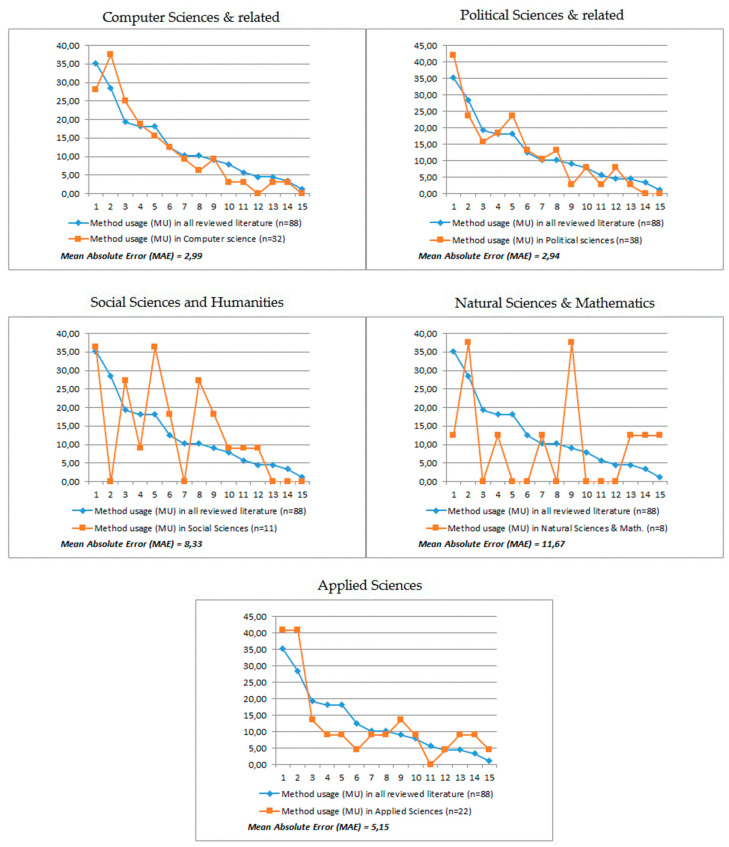
Method usage within the research area groups compared to the reviewed studies. The assignment of particular methods and strategies to the labels numbered from 1 to 15 as described in Table 2.

We also compared the percentage of method usage (MU) in particular research areas to the percentage of MU in all the reviewed studies. This allowed us to see which methods and strategies were used more frequently and which were used less frequently in the examined research areas. Below, in Figure 10, we present the visualization of this comparison. The visualized difference between MU in the whole sample and in particular research areas, from this point forward referred to as the difference in percentage points (DPP). The source data are presented in Table A1 in Appendix B.

The mean absolute error (MAE) analysis has shown that computer science and political sciences are the most characteristic areas of research for issues related to CI and policymaking. As can be seen, in the field of computer science, the most important methods that were used most often in the entire analyzed sample were the analysis of created values (the difference in percentage points, or DPP: +9.09) and the analysis of e-participation process (DPP: +5.68). In turn, the most underrepresented methods were analysis of organizational structure (DPP: −7.10), analysis of impact on policymaking (DPP: −4.83) and state-of-the-art review (DPP: −4.55). On the other hand, in the field of political sciences, as if in opposition to the previous group, an increased interest in analysis of organizatonal structure (DPP: +6.88) was observed, as well as in analysis of collaboration model (DPP: +5.50), whereas low interest in analysis of decision-making (DPP: −6.46) was observed. It is also noticeable that in this group, as in the entire study sample, the analysis of impact on policymaking method is relatively rarely used, which is surprising. When it comes to the research area of the social sciences and humanities (other than political science), we noticed the great popularity of the analysis of participants’ behavior (DPP: +18.18) and the analysis of innovation process (DPP: +17.05), with a complete lack of interest in the analysis of created values. On the other hand, the research conducted within the natural sciences and mathematics was characterized by the little use of the analysis of organizational structure (DPP: −22.73) and the analysis of the e-participation process (DPP: −19.32), but a significantly increased use of the analysis of decision-making process (DPP: +28.41). However, it should be remembered that the studies assigned to areas no. 3 and no. 4 constituted a much smaller sample than those grouped in other areas. Finally, the last presented group of disciplines are applied sciences. In this group, as in computer sciences, the increased use of the analysis of created values (DPP: + 12.50) is observed, and at the same time we see the smaller than in the entire sample, use of the analysis of participants’ behavior (DPP: −9.09), and the analysis of collaboration model (DPP: −9.09).

### 3.4. Methods and Strategies Used in the Most Influential Works and in the Topics of Special Importance

#### 3.4.1. Analysis of the Most Influential Studies

To analyze the most influential studies, we ranked the top 10 articles based on the usage and citation criteria. First, when analyzing the usage criterion, we examined data obtained from the Web from Science: the Since 2013 usage and the 180 Day Usage Count variables. However, we observed that the differences in the top 10 lists generated on their basis were relatively small, so we decided to choose the Since 2013 usage variable for creating the ranking. The results are presented in Appendix C in Table A4.

Secondly, we have prepared a ranking of the top 10 articles based on the criterion of the highest citations (Times Cited, WoS Core). The results are shown below in Appendix C in Table A5.

**Figure 11:**
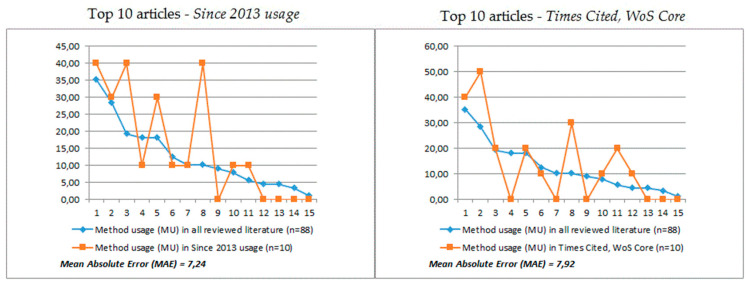
Method usage within the most influential studies compared to all the reviewed literature; 1 to 15 are as described in Table 2.

Finally, we analyzed which methods and strategies of studying CI in policymaking were used in the created sets of the most influential studies. As previously, we compared the percentage of method usage in the most influential studies to the percentage of method usage in all reviewed studies. This allowed us to determine which methods and strategies were used more frequently and which were used less frequently in the examined groups, in a similar way as we did before with research areas. In Figure 11 we present the visualization of this comparison. The source data are presented in Table A2 in Appendix B.

The analysis made it possible to observe interesting similarities and differences between the examined collections of research articles. First, their most common feature was an increased interest in the analysis of innovation process; respectively, DPP +29.77 in the most-read articles group, and DPP +19.77 in the most-cited texts group. Likewise, the analysis of organizational structure is an equally popular method in both groups (DPP +4.77). However, the differences are revealed mainly in the use of analysis of created values: in the group of the most often cited, it is one of the most popular approaches for half of all texts, and DPP +21.59 compared to the use in the entire study sample. However, although it is among the most widely read, this method was not more popular than the entire sample. We have the opposite situation in the case of the analysis of e-participation process: Among the most frequently read texts we can see an increased interest in this method (DPP +20.68), which is not the case with the most often cited texts (DPP +0.68).

#### 3.4.2. The Analysis of Topics of Special Interest

The last stage of our analysis was to examine, within the reviewed literature, the topics of special interest for the research on CI in policymaking. To ensure data triangulation, and to avoid duplicating regularities that were already detected, in the selection of topics we relied on a different method than the one used in the earlier stages of the work. When selecting specific topics for analysis, we relied on monographs concerning issues of collective intelligence and policymaking, published after 1990. The method of selecting topics for analysis is described in Appendix D. The final list of seven topics included: Citizenship, Communities, Consensus, Deliberation, Diversity, Local governance and Urban development, and Open data.

Next, we searched our literature database for the keywords specific to each of these topics. The topic-oriented subgroups of studies were created, based on the occurrence of the related keywords. The results are presented in Table 13.

**Figure 12:**
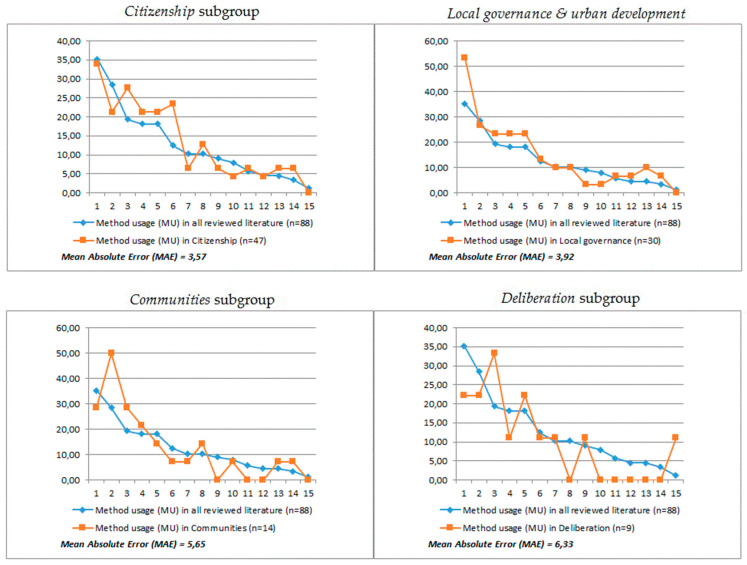
Method usage within the most influential studies compared to all the reviewed studies. The assignment of particular methods and strategies to the labels numbered from 1 to 15 as described in Table 2.

The four most popular topic-oriented subgroups were analyzed in terms of the methods and strategies that were adopted in the conducted research. The aim was to verify to what extent the reviewed literature relates to the examined topics, and what research methods were used in the studies focused on these topics. The results of the analysis are shown in Figure 12. The source data are presented in Table A3 in Appendix B.

## 4. Discussion

The analyses conducted allowed us to conclude that throughout the whole sample the approaches that were most frequently used to study collective intelligence in the domain of policymaking were analysis of the organisational structure and analysis of the created values. Moreover, the analysis of the two most important research areas in which the studies were conducted revealed that the first of these methods is primarily peculiar to political science, and the latter is more common in computer science. Apart from this general observation, we were able to investigate a number of other issues related to the analyzed topic.

We observed that at least since 2015, the topic of CI in policymaking remains a subject of increasing interest among researchers. Although 2017 was the peak of interest, the subsequent years also demonstrated the continued popularity of this issue. Content analysis allowed for the identification of concepts that constituted the most important points of reference in the studies. The dominance of the term crowdsourcing, both in article titles and in author keywords, is noticeable. Due to the fact that this term in its original meaning mainly referred to business projects, we can see that many authors remain rooted to translating patterns developed in the commercial sector into the public sphere. This observation seems to be consistent with the analysis of research methods. The frequent use of analysis of the created values approach is also a common point with commercial projects, in which the direct results of collective effort are one of the primary subjects of interest. In turn, concepts such as the public and government frequently appearing in article abstracts, embedding the research in the political sciences domain. In addition, the KeyWords Plus analysis (based on the literature cited in the analyzed works) shows that the concepts that were most frequently referred to were innovation and participation. Note that the term innovation, in its business sense—being a multi-stage process whereby organisations transform ideas into new/improved products, service or processes [A1]—is now increasingly used in social and political sciences to describe the process of reforming public organizations by opening them to participation [A2], which was also confirmed by our analysis.

Statistical analysis proved that some significant relationships between the research methods can be observed. The negative relationship between the analysis of created values and the analysis of collaboration model is particularly noteworthy. This can be explained by the fact that projects mainly oriented at generating new values are studied in the context of the existing governance framework. The studies on new models of intersectoral collaboration between public and private entities, when the scope of the project extends beyond the structure of one specific organization, require a different approach. The remaining relationships are fairly obvious: A common combination in the reviewed studies was to analyze the behavior and motivation of the participants at the same time. Similarly, it is not surprising that state-of-art-review and categorization of implemented projects were linked. The observed positive relationship between the analysis of created values and the 180 Day Usage Count also led to interesting observations. It can be concluded that the use of the analysis of created values method translates into increased popularity among readers. On the other hand, we can see that studies based on this method result in texts with fewer pages, which makes them more accessible to readers.

The analysis of research areas in which the studies were conducted points to the conclusion that the number and diversity of the scientific disciplines covered by the review is growing year by year. References to CI and policymaking appear in more and more specialized works related to the implementation of public policies. It shows that reflections on CI in policymaking have moved from general considerations to the application of solutions in specific domains of public policy. Secondly, the analysis of the number of studies appearing yearly in research area groups confirmed that researchers tend to be less interested in technological aspects of projects (the computer science and related group), and more in the implementation of these projects in diverse areas of administration, and in the public sphere (the political sciences and related group). As we have already emphasized, the patterns of analysis borrowed from business projects (i.e., created value analysis) were the leading methods of study in computer science. At the same time, the analysis conducted from an organizational perspective was characteristic of contemporary governance studies on CI. However, the low popularity of the analysis of the impact of AI algorithms approach was surprising. It seems that CI studies are still conducted almost entirely separately from AI studies. Despite the fact that the combination of AI and CI has been recently proposed as one of the most important topics of research, for example, in the report *Identifying Citizens’ Needs by Combining AI and CI* [68] or in the works of G. Mulgan [69], it looks like this demand has not yet been answered. The relatively low popularity of the analysis of the impact on policymaking is also puzzling. It can be concluded that the practical function of CI in policymaking is often reduced to fitting CI projects into the existing administrative structure, or on increasing efficiency in achieving goals formulated at the political level, whereas actual shaping of public policy agendas is still rare. Nevertheless, the observed decline in the popularity of the analysis of organisational structure approach may herald some changes.

Research into created values is not the only approach that stands out in computer science. We also notice the popularity of studies on the e-participation processes, focused on engaging wide audiences in policymaking, which is promising in the context of future research. It is also interesting that in the political sciences, apart from research on the organizational structure, there is a significant interest in collaboration models. Reflecting on the cooperation of different types of partners, achieving mutual benefits seems to be a promising model for the future shape of policymaking.

A review of the most influential articles, taking into account both their use and citations, allowed their specific features to be captured. The innovation analysis was a particularly popular research approach in this group. Our observation may be an indication for future research that including the analysis of project innovativeness in the planned works may contribute to increased interest in research results. However, as in the other analyzed subgroups, the number of studies tracking the actual impact of CI projects on shaping public policies was still unexpectedly low. Conversely, the analysis of the e-participation process enjoys increased popularity in this group, although only among the frequently read, though not among the most cited articles. We also noted that the articles relating to user behavior were underrepresented in this group. 

Finally, the analysis of the selected topics of interest showed that the most popular concept in our sample was citizenship, and studies using this term were often associated with the method of analyzing the motivations of participants. This is in line with postulated changes in the relationship between citizens and the state, as proposed by Noveck [67] and others. The government is expected to transform from an authoritative problem-solving center into an arbiter, inviting the citizens to jointly seek the best solutions. Putting the citizens at the center of interest and studying their motivations enhances their role as active participants in the online public sphere. Another very popular concept in the analyzed sample was local governance. References to this topic could be found in over 34% of the reviewed studies. The analysis showed that cities, as well as communities (both local and based on interests), have become the main field of implementation of CI projects in the public space. In the case of cities, the organizational structure of projects was the main method of study, and in the case of communities, the values they produce were more important. It was also noted that topics with a deep theoretical foundation, such as diversity or consensus, were still not very popular among the analyzed works, which may be related to their relatively low applicability to the leading topics of citizenship and local governance.

## 5. Conclusions

Opening policymaking tasks to public participation has become one of the major trends in public policy in recent years. Regarding the 2030 Agenda for Sustainable Development, approved by United Nations Member States in 2015, “responsive, inclusive, participatory and representative decision-making at all levels” is one of the adopted strategic goals for the future [70]. The role of governments is substantially changing, and the emergence of new and complex social problems requires looking for new ways to collaborate in making public decisions with non-governmental actors, and with self-organized communities. For this reason, there is a need to constantly review the existing research on collective intelligence in the domains of public policy and the methods of studying this topic, which may contribute to the better planning of future implementations.

In the present study we made an attempt to identify which methods and strategies have been used so far for researching CI in policymaking. To answer Research Question 1, we conducted a systematic literature review following the PRISMA methodology, supplemented by an analysis of article titles, abstracts and keywords, the yearly number of publications, as well as qualitative research based on the grounded theory method. We identified 15 methods in the analyzed sample. The analysis of the organizational structure and analysis of the created values approaches proved to be the most frequently used approaches.

Considering Research Question 2, the analysis of statistical dependencies allowed us to identify several positive and negative correlations between research methods and between research methods and other variables (especially usage count, as well as the number of pages).

Considering Research Question 3, we found that studies were conducted mainly in computer sciences and political sciences, with the latter group, though initially less numerous, becoming dominant in recent years. We also identified which research methods were more common and which were less common in particular research areas.

Finally, considering Research Question 4, it is possible to conclude that the most influential, i.e., the most cited and the most popular articles, differed from typical studies in terms of the research methods used. A similar phenomenon occurred in relation to groups of articles built around topics of special importance.

The authors hope that by publishing this article they contributed to the systematization of knowledge about studies on collective intelligence in policymaking, showing in which areas the research has been conducted and which methods have been used for this purpose. In addition to identifying the most popular methods, we have attempted to identify the underrepresented approaches, which are promising for the future development of these studies. The present study differs significantly from the studies that were conducted in the past. None of the literature reviews on CI and public policymaking have so far developed a comprehensive list of analytical methods and approaches used in this type of research. For example, Prpić et al. presented the status of research focusing on three selected policy crowdsourcing techniques (virtual labor markets, tournament crowdsourcing, open collaboration), to compare them to the different stages of the policy cycle [37]; Liu et al. synthesized prior research and practices mainly to provide practical lessons for designing new projects in the public sector [52] and Linders focused on classifying citizen co-production initiatives [54]. As our review shows, some types of research have so far been extremely rare. For example, only one study in the analyzed sample concerned organizational learning, and yet, according to studies conducted by Mulgan [4] and Malone [71], it is one of the most important elements involved in collective intelligence. The state of research on the impact of CI in shaping public policy agendas, and on the use of AI algorithms in implemented projects also seems insufficient. We trust that by indicating the areas in which research is still limited, we will contribute to the better quality of future studies.

## Figures and Tables

**Table 1:** Rankings of top 10 concepts based on: (**a**) article titles, (**b**) article abstracts, (**c**) author keywords, (**d**) KeyWords Plus.

(a)		(b)	
Top 10 Concepts in ARTICLE TITLES		Top 10 Concepts in ARTICLE ABSTRACTS	
Concept	Number of Occurrences	Concept	Number of Occurrences
Crowdsourcing	24	Public	153
Open	16	Crowdsourcing	125
Public	16	Government	84
Social	13	Data	82
Innovation	11	Social	79
Case	10	Open	78
Government	9	Innovation	76
Participation	9	Research	64
Online	9	Policy	63
Policy	9	Online	51
**(c)**		**(d)**	
**Top 10 concepts in AUTHOR KEYWORDS**		**Top 10 concepts in KEYWORDS PLUS**	
**Concept**	**Number of occurrences**	**Concept**	**Number of occurrences**
Crowdsourcing	50	Participation	14
Open	21	Innovation	14
Public	21	Media	9
Policy	19	Social	9
Government	16	Coproduction	8
Innovation	16	Government	8
Social	14	E-Government	7
Participation	11	Information	6
Data	10	Democracy	6
Democracy	10	Engagement	6

**Table 2:** Methods and strategies of studying CI in policymaking identified in the reviewed literature.

No.	Method of Studying CI	Description	Literature	No. of Assigned Articles
1.	Analysis of organisational structure/design (RM1)	The studies conducted from organisational perspective. Analysis covers the structures that facilitate the coordination and implementation of rules, resources, technologies, stakeholders, and particular tasks in specific projects or initiatives of open policymaking. These studies present the systems for accomplishing and connecting the activities that occur within examined work organisations, enabling the emergence of CI.	[37,38,39]	31
2.	Analysis of created values (RM2)	The studies aim to answer the question: What kind of valuable results were produced in the analysed projects? The analysis of outputs, concerning that they are more valuable, than the inputs, is conducted. For example: epistemic, democratic and economic values in increasing the quality of public service provision can be analysed.	[40,41]	25
3.	Analysis of e-participation process (RM3)	The aim of the studies is an analysis of factors that influence the technologically supported participation, or e-participation, which can be defined as *participation in societal democratic and consultative processes mediated by information and communication technologies, primarily the internet* [13] or as *the use of information technologies to engage in discourse among citizens and between citizens and elected or appointed officials over public policy issues* [41].	[13,42]	17
4.	Analysis of participants’ behaviour (RM4)	The studies aim to answer the question: What sort of various activities was performed by the users of the examined policymaking platforms and initiatives, what types of operations did they engage in, and how was it related to their individual characteristics.	[43]	16
5.	Analysis of collaboration model (RM5)	It is investigated what forms of collaboration between governmental and non-governmental entities occur in the area under study, and what factors influence its facilitation.	[44]	16
6.	Analysis of participants’ motivations (RM6)	The studies focus on understanding the participants’ motivations to engage in open policymaking projects.	[43,45]	11
7.	Analysis of communication model (RM7)	Analyses of the communication processes, information exchange, establishing information channels between public and civic entities, extraction of valuable information, and the mutual understanding of the content provided are performed.	[46,47]	9
8.	Analysis of innovation process (RM8)	Investigating the critical aspects of innovation process in the studied policymaking projects and initiatives/. The studies aim to answer the following questions: what influences innovation capacity, how to stimulate pro-innovative behaviour, what are the potential positive and negative impacts of the outcomes of the innovation processes.	[44,48,49]	9
9.	Analysis of decision-making process (RM9)	The studies aim to answer the question: How collective intelligent policy decisions are made, and what affects the quality of the decision-making process. The analysis of processes, sub-processes, and data related to collective decision-making is conducted.	[50,51]	8
10.	Analysis of the impact on policymaking (RM10)	The studies present the observed impact of the analysed projects on creating public policies, assess the significance of this impact and factors that influenced it.	[52,53]	7
11.	Categorization of the implemented projects (RM11)	Typologies of various governmental or non-governmental initiatives and projects, engaging citizens in policymaking in a model that consider the emergence of collective intelligence, are presented.	[54]	5
12.	State-of-the-art review (RM12)	The state of research and practices are presented in these studies in a cross-sectional manner. The studies focus on collecting, categorizing and situating the previously published research and practices in the field, coming from the multiple disciplines.	[37,53]	4
13	Analysis of platform usability (RM13)	These studies aim on understanding the structure of policy-oriented websites, their functions, interfaces and the contents; simplicity of use; the site navigation, and the ability of users to control their activities.	[55,56]	4
14	Analysis of the impact of AI algorithms (RM14)	The aim of these studies is an analysis of the possibilities of using AI techniques in CI processes occurring in policymaking initiatives, and the possible effects of their operation.	[57]	3
15.	Analysis of organisational learning (RM15)	The studies focus on organisational learning, as the process of creating, retaining, and transferring knowledge within an policymaking organisation, when an organisation improves over time as it gains experience.	[58]	1

**Table 3:** *p*-values from Pearson’s Chi-squared test of independence applied to each pair of research method variables (where, for example, RM1 stands for Research Method 1). The assignment of particular methods and strategies to the labels numbered from RM1 to RM15 is described in Table 2.

	RM 1	RM 2	RM 3	RM 4	RM 5	RM 6	RM 7	RM 8	RM 9	RM 10	RM 11	RM 12	RM 13	RM 14	RM 15
**RM** **1**	0.000														
**RM** **2**	0.371	0.000													
**RM** **3**	0.089	0.273	0.000												
**RM** **4**	0.089	**0.030**	0.143	0.000											
**RM** **5**	0.430	**0.005**	0.524	0.434	0.000										
**RM** **6**	0.933	**0.026**	0.919	**0.000**	1.000	0.000									
**RM** **7**	0.541	0.225	0.816	0.562	0.214	0.894	0.000								
**RM** **8**	0.900	0.225	0.261	0.136	**0.002**	0.231	0.285	0.000							
**RM** **9**	0.158	0.062	0.608	0.600	0.662	1.000	0.824	0.317	0.000						
**RM** **10**	0.227	0.377	0.177	0.781	0.781	0.882	0.712	0.712	0.383	0.000					
**RM** **11**	0.090	0.668	0.260	0.278	0.278	0.384	0.437	0.437	0.467	0.498	0.000				
**RM** **12**	0.131	0.197	0.317	0.335	0.335	0.439	0.490	0.490	0.517	0.547	**0.000**	0.000			
**RM** **13**	0.661	0.877	0.317	0.717	0.091	0.439	0.318	0.490	0.257	0.547	0.615	0.655	0.000		
**RM** **14**	0.944	0.267	0.388	0.406	0.489	0.505	0.179	0.552	0.137	0.604	0.665	0.701	**0.000**	0.000	
**RM** **15**	0.458	0.526	0.623	0.635	0.635	0.704	0.734	0.734	0.750	0.767	0.805	0.826	0.826	0.850	0.000

**Table 4:** Contingency tables of Pearson’s Chi-squared test of independence for the variables with statistically significant dependency. The assignment of particular methods and strategies to the labels numbered from RM1 to RM15 is described in Table 2.

	**Research method 4**				**Research method 5**		
**Research method 2**	0	1	Sum	**Research method 2**	0	1	Sum
0	48	15	63	0	16	47	63
1	24	1	25	1	0	25	25
Sum	72	16	88	Sum	16	72	88
	**Research method 6**				**Research method 6**		
**Research method 2**	0	1	Sum	**Research method 4**	0	1	Sum
0	52	11	63	0	68	4	72
1	25	0	25	1	9	7	16
Sum	77	11	88	Sum	77	11	88
	**Research method 8**				**Research method 12**		
**Research method 5**	0	1	Sum	**Research method 11**	0	1	Sum
0	5	11	16	0	82	1	83
1	4	68	72	1	2	3	5
Sum	9	79	88	Sum	84	4	88
	**Research method 14**						
**Research method 13**	0	1	Sum				
0	83	1	84				
1	2	2	4				
Sum	85	3	88				

**Table 5:** *p*-values from Yates’s Chi-squared test of independence.

Relationship between	RM2 & RM4	RM2 & RM5	RM2 & RM6	RM4 & RM6	RM5 & RM8	RM11 & RM12	RM13 & RM14
*p*-value	0.062	0.013	0.061	0.00016	0.009	5.05 × 10^−7^	0.00012

**Table 6:** *p*-values of the Shapiro–Wilk test of normality applied for variables: Cited Reference Count, Times Cited WoS Core, Times Cited All Databases, 180 Day Usage Count, Since 2013 Usage Count, Number of Pages.

Cited Reference Count	Times Cited WoS Core	Times Cited All Databases
8.85 × 10^−5^	1.37 × 10^−18^	1.28 × 10^−18^
180 Day Usage Count	Since 2013 Usage Count	Number of Pages
6.3 × 10^−16^	6.78 × 10^−16^	0.0307

**Table 7:** Qualifying intervals for variables.

	Low		Medium		High	
	Range	N	Range	N	Range	N
**Cited Reference Count**	0–40.71	29	40.72–59.42	29	59.43–173	30
**Times Cited WoS Core**	0–3	34	4–11	27	12–502	27
**Times Cited All Databases**	0–3	33	4–11	27	12–512	28
**180 Day Usage Count**	0	30	1–2	38	3–39	20
**Since 2013 Usage Count**	0–8	33	9–23.42	25	23.43–454	30
**Publication Year**	2012–2014	10	2015–2017	38	2018–2020	40
**Number of Pages**	4–13	31	14–19	29	20–34	28

**Table 8:** *p*-values from the Pearson’s Chi-squared test of independence (where CRC stands for Cited Reference Count, CW for Times Cited WoS Core, CA for Times Cited All Databases, 180U for 180 Day Usage Count, 2013U for Since 2013 Usage Count, PY for Publication Year and NoP for Number of Pages).

	RM 1	RM 2	RM 3	RM 4	RM 5	RM 6	RM 7	RM 8	RM 9	RM 10	RM 11	RM 12	RM 13	RM 14	RM 15
**CRC**	0.929	0.750	0.149	0.376	0.554	0.197	0.246	0.211	0.382	0.329	0.071	**0.017**	0.338	**0.042**	0.357
**CW**	0.905	0.232	0.156	0.775	0.456	0.906	0.188	0.511	0.503	0.586	0.340	0.124	0.892	0.085	0.319
**CA**	0.954	0.298	0.415	0.748	0.454	0.897	0.181	0.563	0.465	0.560	0.052	0.141	0.869	0.075	0.319
**180U**	0.398	**0.049**	0.983	0.113	0.879	0.157	0.633	0.054	0.944	0.407	0.959	0.361	0.925	0.447	0.376
**2013U**	0.239	0.726	0.877	0.929	0.850	0.823	0.505	0.780	0.258	0.939	0.907	0.864	0.318	0.263	0.430
**PY**	0.872	0.930	0.623	0.473	0.545	0.087	0.798	0.505	0.944	0.159	0.484	0.398	0.763	0.155	0.545
**NoP**	0.848	**0.005**	0.848	0.512	0.479	**0.038**	0.272	0.704	0.656	0.543	0.812	0.758	0.758	**0.042**	0.357

**Table 9:** Contingency tables of the Pearson’s Chi-squared test of independence for the variables with statistically significant dependency.

	**180 Day Usage Count**					**Number of Pages**			
**Research method 2**	High 180U	Medium 180U	Low 180U	Sum	**Research method 2**	4–13	14–19	20–34	Sum
0	11	32	20	63	0	16	22	25	63
1	9	6	10	25	1	15	7	3	25
Sum	20	38	30	88	Sum	31	29	28	88
	**Number of Pages**					**Cited Reference Count**			
**Research method 6**	4–13	14–19	20–34	Sum	**Research method 12**	Low CR	High CR	Medium CR	Sum
0	30	26	21	77	0	29	26	29	84
1	1	3	7	11	1	0	4	0	4
Sum	31	29	28	88	Sum	29	30	29	88
	**Cited Reference Count**					**Number of Pages**			
**Research method 14**	Low CR	High CR	Medium CR	Sum	**Research method 14**	4–13	14–19	20–34	Sum
0	26	30	29	85	0	31	26	28	85
1	3	0	0	3	1	0	3	0	3
Sum	29	30	29	88	Sum	31	29	28	88

**Table 10:** *p*-values from the Fisher exact test of independence (where RM6 stands for Research method 6, NoP for number of Pages and CRC for cited reference count).

Relationship between	RM6 & NoP	RM14 & NoP	RM14 & CRC	RM12 & CRC
***p*-value**	0.042	0.063	0.067	0.032

**Table 11:** *p*-values from the Chi-squared test of independence (where NoM stands for Number of Methods, CRC for Cited Reference Count, CW for Times Cited WoS Core, CA for Times Cited All Databases, 180U for 180 Day Usage Count, 2013U for Since 2013 Usage Count, PY for Publication Year and NoP for Number of Pages).

Relationship between	NoM & CRC	NoM & CW	NoM & CA	NoM & 180U	NoM & 2013U	NoM & PY	NoM & NoP
***p*-value**	0.461	0.681	0.773	0.773	0.970	0.068	0.856

**Table 12:** Research area groups, grouping the WoS research areas, within which the studies on CI in policymaking were conducted in the 2012–2020 period.

Research Area Group (RAG)	WoS Research Areas Included	The Total Number of Studies in 2012–2020
** Computer Science,** **Information Science** **and related **	Computer Science, Information Science & Library Science, Telecommunications, Medical Informatics.	32
**Political Sciences** **and related**	Public Administration, International Relations, Government & Law, Communication, Public, Environmental & Occupational Health.	38
**Humanities** **and Social Sciences,** **other than** **Political Sciences**	Anthropology, Sociology, Psychology, History, Cultural Studies, Education & Educational Research, Arts & Humanities—Other Topics, Social Issues, Urban Studies, Social Sciences—Other Topics.	11
**Natural Sciences** **& Mathematics**	Mathematics, Physics, Physical Geography, Chemistry, Neurosciences & Neurology, Environmental Sciences & Ecology.	8
**Applied Sciences**	Engineering, Health Care Sciences & Services, Business & Economics, Biodiversity & Conservation, Operations Research & Management Science, Science & Technology—Other Topics, Remote Sensing, Forestry	22

**Table 13:** Saturation of the analyzed research studies with selected topics of interest.

Concept	Number of Studies Where the Concept Appeared	References in Monographic Publications
Citizenship	47	[61,62,63,64]
Local governance & Urban development	30	[2,63,64,65]
Communities	14	[2,62,64]
Deliberation	9	[61,62,64,65,66,67]
Open data	7	[64,65]
Diversity	5	[2,61,63,66]
Consensus	5	[61,62,66]

## Data Availability

The data presented in this study are available on request from the corresponding author.

## Appendix B

**Table A1:** Methods and strategies of studying CI in policymaking used in each particular research area group (RAG), compared to all the reviewed studies. NoS stands for number of studies, in which the particular research method is used; method usage (MU) indicates the percentage of studies in which the research method was used; DPP stands for the difference in percentage points between MU in this group and MU in all the reviewed studies; MAE stands for the mean absolute error for the analyzed group. The assignment of particular methods and strategies to the labels numbered from 1 to 15 is described in Table 2.

	Research Methods & Strategies (RM)														
	1	2	3	4	5	6	7	8	9	10	11	12	13	14	15
**All reviewed literature** **(n-88)**															
NoS	31	25	17	16	16	11	9	9	8	7	5	4	4	3	1
Method Usage (MU)	35.23	28.41	19.32	18.18	18.18	12.50	10.23	10.23	9.09	7.95	5.68	4.55	4.55	3.41	1.14
**RAG 1: Computer Science & related (n = 32)**															
NoS	9	12	8	6	5	4	3	2	3	1	1	0	1	1	0
Method Usage (MU)	28.13	37.50	25.00	18.75	15.63	12.50	9.38	6.25	9.38	3.13	3.13	0.00	3.13	3.13	0.00
DPP	−7.10	9.09	5.68	0.57	−2.56	0.00	−0.85	−3.98	0.28	−4.83	−2.56	−4.55	−1.42	−0.28	−1.14
Mean Absolute Error (MAE)	2.99														
**RAG 2: Political Sciences & related (n = 38)**															
NoS	16	9	6	7	9	5	4	5	1	3	1	3	1	0	0
Method Usage (MU)	42.11	23.68	15.79	18.42	23.68	13.16	10.53	13.16	2.63	7.89	2.63	7.89	2.63	0.00	0.00
DPP	6.88	−4.72	−3.53	0.24	5.50	0.66	0.30	2.93	−6.46	−0.06	−3.05	3.35	−1.91	−3.41	−1.14
Mean Absolute Error (MAE)	2.94														
**RAG 3: Social Sciences & Humanities (n = 11)**															
NoS	4	0	3	1	4	2	0	3	2	1	1	1	0	0	0
Method Usage (MU)	36.36	0.00	27.27	9.09	36.36	18.18	0.00	27.27	18.18	9.09	9.09	9.09	0.00	0.00	0.00
DPP	1.14	−28.41	7.95	−9.09	18.18	5.68	−10.23	17.05	9.09	1.14	3.41	4.55	−4.55	−3.41	−1.14
Mean Absolute Error (MAE)	8.33														
**RAG 4: Natural Sciences & Mathematics (n = 8)**															
NoS	1	3	0	1	0	0	1	0	3	0	0	0	1	1	1
Method Usage (MU)	12.50	37.50	0.00	12.50	0.00	0.00	12.50	0.00	37.50	0.00	0.00	0.00	12.50	12.50	12.50
DPP	−22.73	9.09	−19.32	−5.68	−18.18	−12.50	2.27	−10.23	28.41	−7.95	−5.68	−4.55	7.95	9.09	11.36
Mean Absolute Error (MAE)	11.67														
**RAG 5: Applied Sciences (n = 22)**															
NoS	9	9	3	2	2	1	2	2	3	2	0	1	2	2	1
Method Usage (MU)	40.91	40.91	13.64	9.09	9.09	4.55	9.09	9.09	13.64	9.09	0.00	4.55	9.09	9.09	4.55
DPP	5.68	12.50	−5.68	−9.09	−9.09	−7.95	−1.14	−1.14	4.55	1.14	−5.68	0.00	4.55	5.68	3.41
Mean Absolute Error (MAE)	5.15														

**Table A2:** Methods and strategies of studying CI in policymaking used in the most influential studies, compared to all reviewed studies. NoS stands for the number of studies in which the particular research method was used; method usage (MU) stands for the percentage of studies in which the research method was used; DPP stands for the difference in percentage points between MU in this group and MU in all reviewed studies; MAE stands for the mean absolute error for the analyzed group. The assignment of particular methods and strategies to the labels numbered from 1 to 15 is described in Table 2.

		Research Methods & Strategies (RM)													
	1	2	3	4	5	6	7	8	9	10	11	12	13	14	15
**All reviewed literature** **(n-88)**															
NoS	31	25	17	16	16	11	9	9	8	7	5	4	4	3	1
Method Usage (MU)	35.23	28.41	19.32	18.18	18.18	12.50	10.23	10.23	9.09	7.95	5.68	4.55	4.55	3.41	1.14
**Top 10 articles, according to usage criterion—*Since 2013 usage*** ** (n = 10)**															
NoS	4	3	4	1	3	1	1	4	0	1	1	0	0	0	0
Method Usage (MU)	40.00	30.00	40.00	10.00	30.00	10.00	10.00	40.00	0.00	10.00	10.00	0.00	0.00	0.00	0.00
DPP	4.77	1.59	20.68	−8.18	11.82	−2.50	−0.23	29.77	−9.09	2.05	4.32	−4.55	−4.55	−3.41	−1.14
Mean Absolute Error (MAE)	7.24														
**Top 10 articles, according to citation criterion—*Times Cited, WoS Core*** ** (n = 10)**															
NoS	4	5	2	0	2	1	0	3	0	1	2	1	0	0	0
Method Usage (MU)	40.00	50.00	20.00	0.00	20.00	10.00	0.00	30.00	0.00	10.00	20.00	10.00	0.00	0.00	0.00
DPP	4.77	21.59	0.68	−18.18	1.82	−2.50	−10.23	19.77	−9.09	2.05	14.32	5.45	−4.55	−3.41	−1.14
Mean Absolute Error (MAE)	7.97														

**Table A3:** Methods and strategies of studying CI in policymaking used in the subgroups of studies based on selected topics of interest. NoS stands for number of studies in which the particular research method is used; method usage (MU) stands for the percentage of studies in which the research method was used; DPP stands for the difference in percentage points between MU in this group and MU in all reviewed studies; MAE stands for the mean absolute error for the analyzed group. The assignment of particular methods and strategies to the labels numbered from 1 to 15 is described in Table 2.

		Research Methods & Strategies (RM)													
	1	2	3	4	5	6	7	8	9	10	11	12	13	14	15
**All reviewed literature** **(n-88)**															
NoS	31	25	17	16	16	11	9	9	8	7	5	4	4	3	1
Method Usage (MU)	35.23	28.41	19.32	18.18	18.18	12.50	10.23	10.23	9.09	7.95	5.68	4.55	4.55	3.41	1.14
***Citizenship* subgroup** ** (n = 47)**															
NoS	16	10	13	10	10	11	3	6	3	2	3	2	3	3	0
Method Usage (MU)	34.04	21.28	27.66	21.28	21.28	23.40	6.38	12.77	6.38	4.26	6.38	4.26	6.38	6.38	0.00
DPP	−1.18	−7.13	8.34	3.09	3.09	10.90	−3.84	2.54	−2.71	−3.70	0.70	−0.29	1.84	2.97	−1.14
Mean Absolute Error (MAE)	3.57														
***Local governance*** ***& urban development* subgroup** ** (n = 30)**															
NoS	16	8	7	7	7	4	3	3	1	1	2	2	3	2	0
Method Usage (MU)	53.33	26.67	23.33	23.33	23.33	13.33	10.00	10.00	3.33	3.33	6.67	6.67	10.00	6.67	0.00
DPP	18.11	−1.74	4.02	5.15	5.15	0.83	−0.23	−0.23	−5.76	−4.62	0.98	2.12	5.45	3.26	−1.14
Mean Absolute Error (MAE)	3.92														
***Communities*** **subgroup** ** (n = 14)**															
NoS	4	7	4	3	2	1	1	2	0	1	0	0	1	1	0
Method Usage (MU)	28.57	50.00	28.57	21.43	14.29	7.14	7.14	14.29	0.00	7.14	0.00	0.00	7.14	7.14	0.00
DPP	−6.66	21.59	9.25	3.25	−3.90	−5.36	−3.08	4.06	−9.09	−0.81	−5.68	−4.55	2.60	3.73	−1.14
Mean Absolute Error (MAE)	5.65														
***Deliberation*** **subgroup** ** (n = 9)**															
NoS	2	2	3	1	2	1	1	0	1	0	0	0	0	0	1
Method Usage (MU)	22.22	22.22	33.33	11.11	22.22	11.11	11.11	0.00	11.11	0.00	0.00	0.00	0.00	0.00	11.11
DPP	−13.01	−6.19	14.02	−7.07	4.04	−1.39	0.88	−10.23	2.02	−7.95	−5.68	−4.55	−4.55	−3.41	9.97
Mean Absolute Error (MAE)	6.33														

## Appendix C

**Table A4:** Ranking of top 10 articles, according to the usage criterion (Since 2013 usage).

Authors (Year)	Title	Research Area Group	Research Method	Since 2013 Usage	180 Day Usage Count	Times Cited, WoS Core	Times Cited/Year
Linders, D. (2012)	From e-government to we-government: Defining a typology for citizen coproduction in the age of social media	Computer Science & related	2, 10	454	39	502	55.78
Mergel, I.; Desouza, K.C. (2013)	Implementing Open Innovation in the Public Sector: The Case of Challenge.gov	Political Sciences & related	5, 8	192	9	128	16
Diaz-Diaz, R.; Perez-Gonzalez, D. (2016)	Implementation of Social Media Concepts for e-Government: Case Study of a Social Media Tool for Value Co-Creation and Citizen Participation	Computer Science & related; Applied Sciences	1, 2, 3	165	14	17	3.4
Almirall, E.; Lee, M.; Majchrzak, A. (2014)	Open innovation requires integrated competition-community ecosystems: Lessons learned from civic open innovation	Applied Sciences	1, 2, 8, 12	161	4	68	9.71
Mergel, I. (2015)	Opening Government: Designing Open Innovation Processes to Collaborate With External Problem Solvers	Computer Science & related; Humanities & Social Sciences	5, 8	112	1	42	7
Wijnhoven, F.; Ehrenhard, M.; Kuhn, J. (2015)	Open government objectives and participation motivations	Computer Science & related	3, 6	93	2	76	12.67
Mergel, I. (2018)	Open innovation in the public sector: drivers and barriers for the adoption of Challenge.gov	Applied Sciences; Political Sciences & related	8	86	14	38	12.67
Lampe, C.; Zube, P.; Lee, J.; Park, C.H.; Johnston, E. (2014)	Crowdsourcing civility: A natural experiment examining the effects of distributed moderation in online forums	Computer Science & related	1, 3	68	1	53	7.57
Lin, Y.L. (2018)	A comparison of selected Western and Chinese smart governance: The application of ICT in governmental management, participation and collaboration	Political Sciences & related; Computer Science & related	1, 5, 7	63	15	11	3.67
Pieper, A.K.; Pieper, M. (2015)	Political participation via social media: A case study of deliberative quality in the public online budgeting process of Frankfurt/Main, Germany 2013	Computer Science & related; Applied Sciences	3	62	1	3	0.5

**Table A5:** Ranking of top 10 articles, according to the citation criterion (*Times Cited, WoS Core)*.

Authors (Year)	Title	Research Area Group	Research Method	Since 2013 Usage	180 Day Usage Count	Times Cited, WoS Core	Times Cited/Year
Linders, D. (2012)	From e-government to we-government: Defining a typology for citizen coproduction in the age of social media	Computer Science & related	2, 10	454	39	502	55.78
Mergel, I.; Desouza, K.C. (2013)	Implementing Open Innovation in the Public Sector: The Case of Challenge.gov	Political Sciences & related	5, 8	192	9	128	16
Wijnhoven, F.; Ehrenhard, M.; Kuhn, J. (2015)	Open government objectives and participation motivations	Computer Science & related	3, 6	93	2	76	12.67
Almirall, E.; Lee, M.; Majchrzak, A. (2014)	Open innovation requires integrated competition-community ecosystems: Lessons learned from civic open innovation	Applied Sciences	1, 2, 8, 12	161	4	68	9.71
Chen, L.J.; Ho, Y.H.; Lee, H.C.; Wu, H.C.; Liu, H.M.; Hsieh, H.H.; Huang, Y.T.; Lung, S.C.C. (2017)	An Open Framework for Participatory PM2.5 Monitoring in Smart Cities	Computer Science & related; Applied Sciences	1, 2	31	4	68	17
Prpić, J.; Taeihagh, A.; Melton, J. (2015)	The Fundamentals of Policy Crowdsourcing	Political Sciences & related	10, 11	8	0	65	10.83
Lampe, C.; Zube, P.; Lee, J.; Park, C.H.; Johnston, E. (2014)	Crowdsourcing civility: A natural experiment examining the effects of distributed moderation in online forums	Computer Science & related	1, 3	68	1	53	7.57
Stritch, J.M.; Pedersen, M.J.; Taggart, G. (2017)	The Opportunities and Limitations of Using Mechanical Turk (MTURK) in Public Administration and Management Scholarship	Computer Science & related	1, 2	22	1	48	12
Charalabidis, Y.; Loukis, E.N.; Androutsopoulou, A.; Karkaletsis, V.; Triantafillou, A. (2014)	Passive crowdsourcing in government using social media	Computer Science & related	2	1	0	45	6.43
Mergel, I. (2015)	Opening Government: Designing Open Innovation Processes to Collaborate With External Problem Solvers	Computer Science & related; Humanities & Social Sciences	5, 8	112	1	42	7

## Appendix D

The monographic publications concerning (fully or partially) the issues of collective intelligence and policymaking published after 1990 were shortlisted. Due to the scarcity of monographic literature, only eight publications were included in this list after the review. On this basis, an initial list of 20 issues was compiled. Then, a questionnaire was conducted in which a group of six social science researchers were invited to assess the significance of the proposed issues. Thus, the final list of seven concepts that were subject to analysis was selected. The final list of seven topics included citizenship, communities, consensus, deliberation, diversity, local governance and urban development, and open data.

**Table A6:** Monographic publications used as initial references to select the topics of special interest.

Author	Title	Reference
Aitamurto, T.	Crowdsourced Off-Road Traffic Law Experiment In Finland	[61]
Landemore, H.	Democratic Reason: Politics, Collective Intelligence, and the Rule of the Many	[62]
Landemore, H.	Open Democracy: Reinventing Popular Rule for the Twenty-First Century	[63]
Levy, P.	Collective Intelligence: Mankind’s Emerging World in Cyberspace	[2]
Noveck, B.S.	Smart Citizens, Smarter State. The Technologies of Expertise and the Future of Governing	[64]
Noveck, B.S.; Harvey, R.; Dinesh, A.	The Open Policymaking Playbook	[65]
Noveck, B.S.; et al.	Crowdlaw for Congress. Strategies for 21st Century Lawmaking	[66]
Ryan, M.; Gambrell, D.; Noveck, B.S.	Using Collective Intelligence to Solve Public Problems	[67]
